# “Frustrated with the whole system”: a qualitative framework analysis of the issues faced by people accessing health services for chronic pain

**DOI:** 10.1186/s12913-022-08946-8

**Published:** 2022-12-31

**Authors:** Ria E. Hopkins, Louisa Degenhardt, Gabrielle Campbell, Sara Farnbach, Natasa Gisev

**Affiliations:** 1 National Drug and Alcohol Research Centre, UNSW Sydney, 22-32 King Street Randwick, Sydney, NSW 2031 Australia; 2 School of Psychology, University of Queensland, Sir Fred Schonell Drive St Lucia, Brisbane, QLD 4072 Australia

**Keywords:** Chronic pain, Chronic non-cancer pain, Health services, Health service access, Qualitative research

## Abstract

**Background:**

Chronic non-cancer pain (CNCP) is complex and often requires multimodal management comprising of both pharmacological and non-pharmacological treatments. To inform delivery of CNCP management, it is important to understand how current health services providing non-pharmacological treatments are accessed by exploring the experiences of people attempting to access services. In doing so, this study sought to explore the underlying drivers of service access barriers.

**Methods:**

This study explored the experiences of Australians accessing services for CNCP using semi-structured telephone interviews undertaken between 01 October 2020 and 31 March 2021. Thematic analysis was guided by Levesque et al.’s 2013 conceptual framework of access to health care, with emerging themes mapped to five dimensions of accessibility and corresponding abilities of consumers: Approachability/Ability to perceive; Acceptability/Ability to seek; Availability and Accommodation/Ability to reach; Affordability/Ability to pay; and Appropriateness/Ability to engage.

**Results:**

The 26 participants (aged 24–78 years, 22 female) reported accessing a range of services including general practitioners (GP), allied health services, and specialised pain clinics, for a variety of conditions. Three themes were mapped to accessibility dimensions (in brackets): *‘GP as guide or gatekeeper’* (Approachability); *‘Outside of my control’* (Availability and Accommodation; Affordability); and *‘Services aren’t always good enough’* (Appropriateness). A fourth identified theme illustrated how participants responded to encountering these barriers: *‘Leading my own pain management’*. Participant experiences suggest problems with the translation of contemporary pain management principles into practice, including continued application of biomedical health models as opposed to the biopsychosocial model, and demonstrate systemic issues with service delivery, including a lack of benchmarking of specialised services.

**Conclusions:**

The identified themes highlight several evidence-to-practice gaps in the delivery of health services for people with CNCP in Australia. To address these gaps, there is a need for improved clinician training, increased investment in specialised pain services, and development of clear primary care pathways for CNCP management for evidence-based multimodal pain management to be accessible and equitable.

**Supplementary Information:**

The online version contains supplementary material available at 10.1186/s12913-022-08946-8.

## Background

Chronic non-cancer pain (CNCP: pain persisting/recurring over three-months not associated with malignancy) is highly prevalent, ranging between 20 to 40% of the population [1–3]. CNCP is a leading cause of disability worldwide [4], burdening individuals and societies through lost wellbeing, healthcare expenditure, and reduced employment [2, 5]. Effective management is essential to improve quality of life and enable engagement in social and employment activities. In order to inform service planning and delivery, it is essential to understand the accessibility of evidence-based CNCP management and potential barriers to service use.

CNCP is complex, and there are multiple modalities for treating pain, underpinned by the biopsychosocial model of health. Hence, in addition to considering biological causes, aspects relating to psychological and social factors, such as attitudes, behaviours, supports, and relationships, should be incorporated into management [6–9]. Treatment guidelines informed by this model emphasise consideration of both pharmacological and non-pharmacological strategies [10–12].

Pharmacological strategics include the use of medicines such as opioid analgesics, which are commonly used [13] and may provide short-term pain severity reductions, though there is limited evidence supporting long-term benefits or effects on functional outcomes [14, 15]. Non-pharmacological treatments include physical therapies and rehabilitation to improve functioning, and psychological and behavioural therapies addressing psychosocial components of pain [16–19]. Combined with pharmacological treatments, these are associated with greater improvements in pain and disability compared to medicines alone [20, 21]. This form of multimodal management may be delivered by a multidisciplinary team of providers including general practitioners (GPs) or primary care physicians, specialists, allied and mental health practitioners. In some jurisdictions, specialised pain clinics provide access to pain specialists and multidisciplinary pain programs [2, 21, 22].

Although guidelines recommend multimodal pain management [10–12], the health services offered and accessed for CNCP are highly variable. Over a four-year period, we previously identified that a cohort of Australians prescribed opioids long-term for CNCP reported more regular and frequent use of medicines compared to non-pharmacological treatments, with past 12-month specialised pain program attendance reported by less than 10% of the cohort [23]. Similar to international reports [24, 25], these findings support assertions that evidence-to-treatment gaps persist globally [26], and that most people with CNCP do not receive evidence-based multimodal management [2, 27]. Australia has a universal health care scheme and it is important to examine barriers to service use in this context. This study explored the experiences of Australians accessing health services for CNCP, with an emphasis on identifying barriers to service access.

## Methods

### Study design and setting

In this qualitative study, semi-structured interviews were conducted with Australian adults living with CNCP, recruited through social media. Findings are reported in line with COnsolidated criteria for REporting Qualitative research (COREQ) [28].

Australia’s universal health care scheme provides subsidised access to GP, specialist, diagnostic, and some allied and mental health services to Australian citizens and permanent residents [29]. Chronic Disease Management arrangements remunerate GPs for coordinating management of chronic conditions and provide subsidised allied health sessions annually to patients [30]. Australians also have the option of accessing private services and may obtain private health insurance; approximately half of the population hold insurance cover for non-hospital services including allied health services [31]. Most Australians have access to a GP and approximately 88% of Australians visited a GP in 2018–19, although access is generally lower in regional and remote areas [32]. Access to specialist services is variable, particularly between urban and regional areas. A review of Australian pain services identified 109 public and private pain services in 2018, with approximately 0.18% of Australians accessing an adult pain service, and considerable disparities in distribution of services across the country and long waiting lists suggesting demand outweighs supply [22].

### Ethics

The study received ethical approval from the University of New South Wales Human Research Ethics Committee (approval no. HC200517). The protocol was also reviewed and approved by the Chronic Pain Australia Research Committee.

### Study sample and recruitment

Convenience sampling was used with the aim of interviewing approximately 30 participants, or until thematic saturation was achieved, which was sufficient to meet the research aims because this study did not seek to generalise to broader populations, but to gain an in-depth understanding of health service accessibility for CNCP. The study was advertised through paid advertisements on Facebook (Facebook, Inc., Menlo Park CA), by key consumer groups: Chronic Pain Australia and Painaustralia, and on the social media pages (Facebook; Twitter (Twitter, Inc., San Francisco CA)) of the research organisation. Participants were invited to contact the research team and to take part in a five-minute screening procedure via telephone to check inclusion criteria. Participants were reimbursed AUD20 for completing the interview.

Participants were eligible to participate if they had been living with pain for over three months, were 18 years or older, and could complete an interview in English unassisted. Exclusion criteria included having any active malignancy or experiencing pain related to previous malignancy, receiving palliative care services, or being unable to provide informed consent, as deemed by the lead researcher (RH). 

### Data collection tool

A semi-structured interview guide (Supplementary File 1) was developed and piloted, with adaptations made to improve interview flow. Participant characteristics were collected including self-reported age, gender identity, pain condition/s, and years lived with pain. Participant’s residential postcode was used to determine whether they lived in a major city, inner or outer regional area, or remote area, using the Australian Statistical Geography Standard [33]. The interview guide covered the following key areas: history of pain condition/s and current and previous pain management and health services accessed. Participants were asked whether they had accessed or attempted to access different services, including allied health services and specialised care including pain services. Depending on responses, participants were asked about barriers to using services, or reasons for not using services. Participants were asked about their experiences seeking and receiving care, including their satisfaction with pain management, information received about services, and whether expectations for pain management were being met.

### Study procedure

Following confirmation of eligibility, participants were invited to complete a one-hour telephone interview. A Participant Information Statement and Consent Form was provided at least 24-h prior to the interview, and participants provided verbal consent prior to commencing. Recruitment commenced 1st October 2020 and ended 31st March 2021, when no new information was being presented. Interviews were undertaken by the lead researcher (RH), recorded using videoconferencing technology, and transcribed verbatim. The lead researcher (RH) maintained memos and interview notes.

### Reflexivity

Interviews were undertaken by the lead researcher (RH), a female doctoral candidate in her thirties with degrees in health science and public health and training in qualitative methods. The interviewer was a person with lived experience of CNCP. The research team included researchers with expertise in pain management, health services research, and qualitative research methodologies. No member of the research team had an existing relationship with participants, who were aware that the study was being undertaken as part of the lead researcher’s thesis exploring health service utilisation for CNCP.

### Theoretical framework

**Fig. 1:**
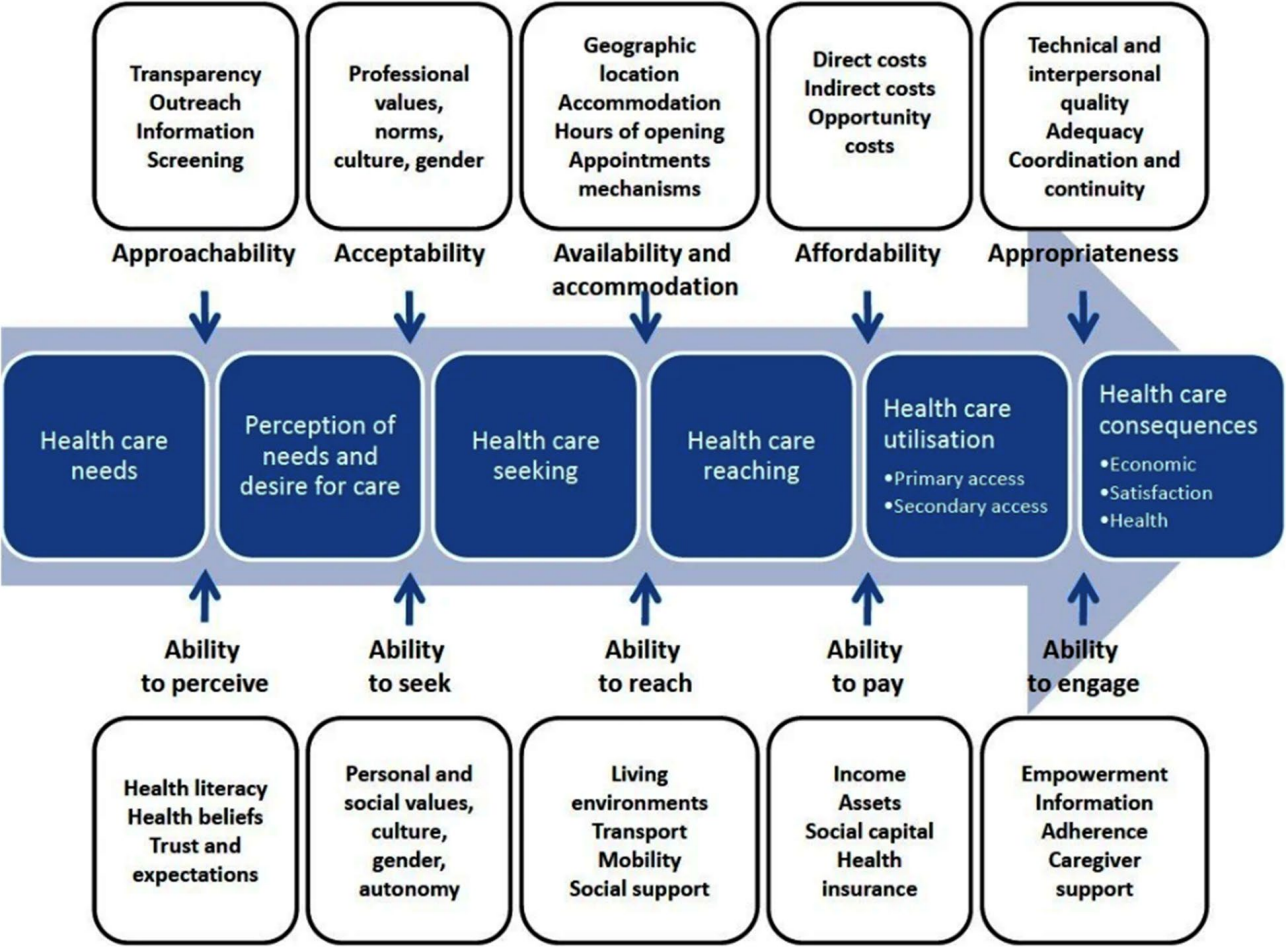
Levesque’s conceptual framework of access to health care [34]. Creative Commons Attribution License: CC BY 2.0

Thematic analysis was undertaken and initially informed by Levesque et al.’s 2013 conceptual framework of access to health care, which defines health care accessibility as *“the opportunity to reach and obtain appropriate health care services in situations of perceived need for care”* [34]. The framework conceptualises health care access beyond simple utilisation of services, framing access as a process beginning with identification of health needs, continuing through to realised consequences of receiving care, with consideration given to the fit between needs and services received (Fig. 1). The framework incorporates five dimensions of accessibility on the part of service providers and five corresponding abilities of the target population: Approachability/Ability to perceive; Acceptability/Ability to seek; Availability and Accommodation/Ability to reach; Affordability/Ability to pay; Appropriateness/Ability to engage.

### Analysis

Data were stored and coded in NVivo v12 Pro (QSR International Pty Ltd., Melbourne VIC), using the thematic analysis process described by Braun and Clarke [35]. Briefly: interviews were listened to and transcripts read several times to become familiar with data. Codes were generated a posteriori using an inductive approach to identify commonalities of meaning. Initial themes were identified and assigned to Levesque’s dimensions, then reviewed against coded extracts and the entire dataset and revised as appropriate until final themes were defined. Although primary responsibility for analysis was held by the lead researcher (RH), peer debriefing was undertaken and regular discussions were held with the project team throughout, with themes developed and refined with team consensus [36].

### Reporting of findings

Key quotes are presented to support thick description and presentation of themes. Participants are identified by participant number (e.g., P1), 10-year age bracket to preserve anonymity, and gender. Where edited for readability, added words are enclosed in [squared brackets] and “…” denotes removed text.

## Results

### Participant characteristics

**Fig. 2:**
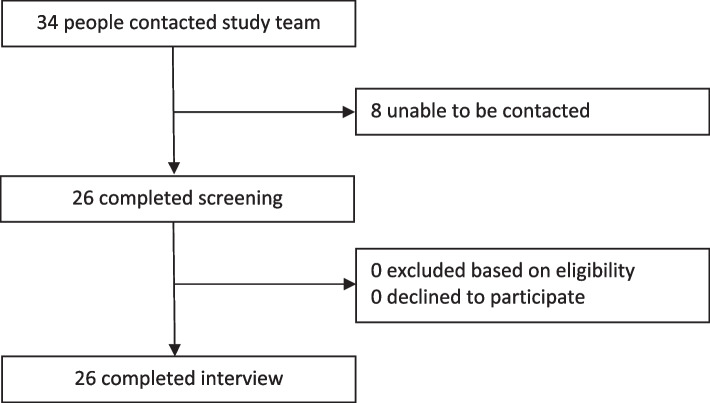
Study recruitment

Overall, 34 people contacted the research team; of these, 26 followed through for screening (Fig. 2). All 26 were eligible for inclusion and proceeded to complete telephone interviews, which ran for an average of 44 min (range 22–71).

**Fig. 3:**
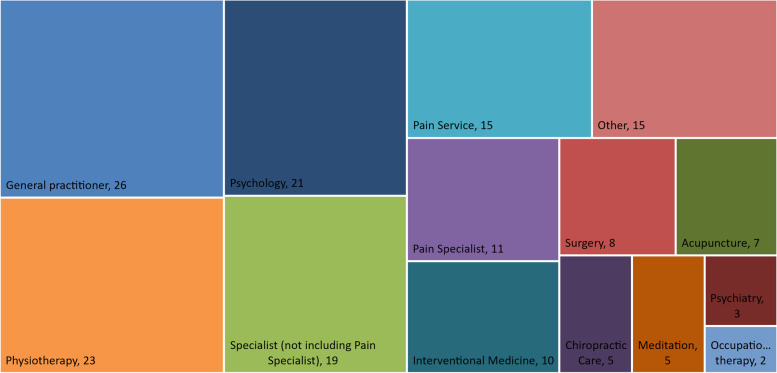
Current and previous health care services reported by participants for chronic non-cancer pain

Participants included 22 women and four men who ranged from 24 to 78 years of age (Table 1). Six participants lived in regional areas. A range of CNCP conditions were reported, with eleven people reporting multiple conditions. Participants reported use of a wide range of services: all were managed by a GP, and a pain specialist or pain clinic had been accessed by 22 participants (Fig. 3).

**Table 1:** Characteristics of included participants

	***N* = 26**
Age, mean (range)	48 (24–78)
Gender, n	
Women	22
Men	4
Non-binary/Other	0
Location, n	
Major city	20
Inner regional	5
Outer regional	1
Remote	0
Years living with pain, mean (range)	17 (1.5–45)
More than one pain condition reported, n	11
Pain conditions reported^a^, n	
Fibromyalgia	7
Spinal cord/disc damage	7
Chronic regional pain syndrome	4
Endometriosis	3
Migraine	3
Psoriatic arthritis	3
Rheumatoid arthritis	3
Nerve damage post-injury	2
Other^b^	4
Unspecified/undiagnosed pain reported, n	5

^a^participants could report more than one condition

^b^includes temporomandibular joint pain, Parkinson’s disease, osteoarthritis, and sciatica, reported by one participant each

### Themes identified

Four major themes were identified, with three matching dimensions in Levesque’s framework (in brackets): ‘*GP as guide or gatekeeper’* (Approachability/Ability to perceive); *‘Outside of my control: External barriers to service access’* (Availability and Accommodation/Ability to reach; Affordability/Ability to pay); and *‘Services aren’t always good enough: There is wide variation in services and quality’* (Appropriateness/Ability to engage)*.* The fourth theme identified, *‘Leading my own pain management:* *The response to accessibility issues’,* illustrated how participants responded to described barriers to service access. Grouping of codes and positioning of themes in relation to Levesque’s framework are demonstrated in Supplementary File 2. There was an absence of codes correlated to the ‘Acceptability/Ability to seek’ dimension, which relates to socio-cultural factors influencing care seeking [34].

#### Theme 1: General practitioner as guide or gatekeeper (Approachability/Ability to perceive)

The first point of contact with the health care system for most participants was a GP. Participants generally expected their GP to act as a touchpoint to the broader health system by recommending potential services or referring them to specialists.

“You expect GPs to sort of be that person who helps coordinate things and your specialist and who sort of oversees your whole care.” P8, 20–30 years old, female

Whether this expectation was met varied. For some, the GP played a critical role in providing guidance and education about potential treatments. They linked participants into and coordinated multidisciplinary management, and participants expressed confidence in their GP’s approach.

“He's [GP] been absolutely fantastic coordinating all the different specialties all over the place to handle everything… I've put myself fully in the hands of the medical practitioners.” P9, 30–40 years old, male.

Other participants reported difficulty finding GPs who proactively managed their pain: they described their symptoms being dismissed or reported a lack of confidence in their GP’s management. Some participants without a clear diagnosis reported that their GP appeared at a loss and described feeling ‘brushed off’, believing not enough was being done to investigate and diagnose their pain.

“I was repetitively complaining about the pain, and I was just getting the response, “Oh, well, there's no medical reason why this could be happening. I don't really know. I can’t really help you any further.”” P25, 20–30 years old, female.

For some, receiving a diagnosis coincided with beginning treatment; for others, treatment did not progress, even after a diagnosis had been made. On receiving a diagnosis of fibromyalgia, one participant reflected:

“I was kind of thrown back out into the world. They were kind of like, “Yep, this is it,” with no real, further direction after that, … It wasn't suggested to me that I find other ways of coping with the pain as well, for example, meditation, psychological health, psychiatry, even physios or physical health.” P25, 20–30 years old, female.

Some participants were pragmatic about the time constraints of GPs, and were understanding that as generalists, GPs often had limited training regarding pain management. However, where a GP had exhausted their knowledge and available treatments, participants expected to be referred to other providers. Failure to do so undermined confidence in the GP, particularly if they were not perceived as being up-front about being unsure of what to do next.

“If they don't understand, say it. Just say, “I actually really don't understand your condition. But I know this person does. So maybe you should talk to this person.” You know, just be open and not go, “Oh, you know, we’re gonna try this, and we're gonna try that”. And not really have any idea.” P3, 40–50 years old, female.

Many participants conducted their own research about their conditions. When they identified a potential treatment or service, participants often required a GP referral, particularly to access government subsidies. Some participants described collaborative relationships with their GPs and were able to make suggestions. Others, however, reported being shut down or having their suggestions dismissed. Participants described having to argue to receive referrals.

“There's still a gatekeeper in between you and that service, which is your GP.” P25, 20–30 years old, female

These responses were often perceived to be power-based, and the overall result was an undermining of the therapeutic relationship between GP and participant.

“It’s like there’s no more suggestions they’ve offered, like I have to do that myself, and when I do bring them in, there’s often that sort of resistance because you’re not the expert, they are.” P8, 20–30 years old, female.

Several participants described being advised against services which are evidence-based and recommended in guidelines, generating confusion, and leaving them feeling like there were no other options.

“I did ask my doctor, my GP, about going to a pain clinic… their response was, “I could refer you, but trust me, you don't want me to send you there because they'll put you on really harsh drugs, which you could get addicted to…”, which was sort of disappointing, because that seemed to be, at that time, all I understood was the option”. P13, 30–40 years old, female.

#### Theme 2: Outside of my control: External barriers limiting access (Availability and Accommodation/Ability to reach; Affordability/Ability to pay)

Once participants received information about and referrals to services, many experienced external barriers to service access.

##### Geographic and physical availability

Issues with physical availability of services were described by participants in both urban and regional areas, with specialist services particularly difficult to access in regional areas. Four of the six regional participants travelled to urban centers for care and experienced added burdens of requiring accommodation and leave from work.

“You’re having to drive long distances, stay overnight, and all that sort of stuff.” P17, 60–70 years old, female

Physical limitations prevented some participants transporting themselves to appointments, while others reported travel exacerbating their pain. For some, the location of services increased the burden associated with seeking care; for others, receiving care was simply not possible.

“I don’t have a license and, being disabled, it makes it a lot more difficult for me to get places… And then the pain services tend to be at the hospital…there is very little public transport that actually allows me to get to any hospital around me.” P15, 20–30 years old, female.

##### Timely access

Following referral, many participants were placed on waiting lists, with waiting times over one year not uncommon for public services. Several participants described being removed from waiting lists without explanation, or being informed services lacked capacity for new patients. There was a perception that referring clinicians and those recommending multidisciplinary care were unaware of the realities of accessing services, leading to frustration as people tried to follow suggestions for management.

“They say, I “just need to go and do this, and multi-this and multi-that,” and I go, “Yeah, but do you know the waiting list times?” “Oh no, we don't.” It's all good to say that in theory but it really, really, believe me, doesn't work that way on the ground and that's the frustration for chronic pain patients when we're told to use all these other modes, you've got to wait.” P20, 50–60 years old, male.

Although shorter waiting times were reported for private services, it was still common to wait months for specialists. Participants also reported long waits between appointments or treatments.

“[It’s] like, “Okay, let’s try this series of nerve blocks. If that doesn’t work, we’ll go to epidurals,” and whatever but, really, there’s six months to nine months in between drinks there, and you just can’t get an appointment.” P12, 40–50 years old, female.

##### Affordability

Participants incurred considerable costs to access services, and those without private insurance described limitations in public cover. Use of government-subsidised Chronic Disease Management arrangements was variable and many were unaware of them. The seven participants who reported using them described the number of subsidised sessions as insufficient for managing chronic conditions.

“[The five sessions were] nice to have, but I’d used them in ten days, ‘cause I was having a really bad patch and needed some intensive work from the physio. So, that left 355 remaining days.” P26, 20–30 years old, female.

Participants holding private insurance often incurred out-of-pocket expenses and similarly found the level of care covered was insufficient.

“They often have a ceiling of $500 anyway [for physiotherapy], and then you have spent that in like three visits, so it’s gone. It’s one or two weeks of visits, not the whole year.” P20, 50–60 years old, male.

With some participants unable to work due to pain, the cost of treatments added to existing financial stress, and health care was often not prioritised. For employed participants, time away from work to attend treatments imposed further burden.

#### Theme 3: Services are not always good enough: There is wide variation in services and quality (Appropriateness/Ability to engage)

Once use of health services was initiated, participants’ experiences of services, and their ability to engage and experience benefits from treatment, varied widely. This was most apparent among descriptions of specialised pain services: most participants had attended a pain specialist or clinic, but there were differences in the care received. For some, the pain clinic was an invaluable ‘one-stop shop’, linking them in with specialists and allied health services, with all providers working according to a coordinated treatment plan.

“I have a multidisciplinary approach. So, I see a pain specialist, who coordinates with a psychologist, my GP. I also have access to a physiotherapist.” P1, 20–30 years old, female

However, some participants were disappointed to find that the pain services they accessed appeared focused solely on providing interventional treatments or reducing opioid medicines, contrary to their expectations of receiving the multidisciplinary care described above.

“His [pain specialist’s] only approach in that two years has been to reduce my medication… I thought I was seeing him because he was going to be working up a whole approach to the whole thing. But his entire thing was just focused on reducing meds.” P5, 60–70 years old, female.

After waiting months to access a public pain service, one participant described being told the service was not appropriate for her because she was not taking opioid medicines at the time of admission.

“The whole clinic is super focused on like opioids and getting you off opioids, and it’s like ‘cause you’re not completely debilitated and on these high medications, like, “Sorry, but there’s nothing we can do for you.”” P8, 20–30 years old, female.

Services were often described as appearing limited to applying the traditional biomedical model of health rather than the biopsychosocial model, with practitioners not necessarily trained to treat CNCP in a contemporary way.

“They’re [physiotherapists] very rudimentary. I don’t think a lot of them can think outside of doing this exercise and that exercise… You’ve got to fit into their box. If you don’t fit into their box, then you’re stuffed.” P11, 50–60 years old, female.

Participants also described difficulties engaging when referred to providers without specific experience or training in the management of CNCP, such as general physiotherapists or psychologists.

“Sports physicians, physios, etcetera, have some experience dealing with people who are in pain, or rehabilitation and things like that. But I think that it's actually a really different skill set to dealing with long-term chronic pain.” P1, 20–30 years old, female.

Service and quality variability were linked with participant satisfaction with pain management. Overall satisfaction often had little relation to the level of pain reported: some participants continued to experience severe pain but reported being satisfied with providers who they perceived to be working proactively with them.

“I’ve been lucky to find a pain care team… I don’t have a successful pain treatment program at the moment, but I do have people that are working with me to identify something that might work.” P18, 30–40 years old, female.

Conversely, dissatisfied participants described treatments which were not patient-centered or personalised, and which did not consider their individual treatment goals.

“All the waiting around, there are 30 people in the waiting room, one after the other seen in the pain management clinic for about five minutes. The nurse comes out, waves them, they’re seen, all a production line.” P24, 60–70 years old, male.

#### Theme 4: Leading my own pain management: The response to accessibility issues

Experiencing accessibility issues had a profound impact on many participants. The overall experience of attempting to access services and treatments was described as long, frustrating, and demeaning. Some participants reacted by disengaging from the health care system and, at time of interview, several participants were not using services beyond visiting their GP for prescriptions.

“I’ve given up with it since 2008,’09. I’ve given up. It’s just about impossible, like I said, especially as a public patient… The system is very discouraging, and it can feel really lonely and difficult.” P25, 20–30 years old, female.

Two participants described their experiences as so distressing that they avoided the health care system entirely.

“I try not to go to the doctor or use any of the services anymore. I think the medical system has caused me more trauma than it has done good for me.” P8, 20–30 years old, female

After a period of disengagement, however, many participants recommenced their efforts to find effective pain management. Often, this re-engagement followed a period of active self-management and was accompanied by a new assertiveness.

“I thought, ‘Stuff this! This is just bullshit! I’m just gonna find a doctor that I can sort this out with.’ I’d already worked out that if this doctor and this rheumatologist weren’t gonna work out for me… I was just gonna flip them and get a new one. And start down that path again.” P17, 60–70 years old, female.

Participants described undergoing a shift from “passive consumer” to active or “working patient” *(P18)*, taking the lead in their health care team. Participants described learning to advocate for the care they believed they should be receiving based on their own research and information gained outside of the system.

“[GPs are] not gonna really go and try and manage it for you. You’ve really got to take charge yourself.” P12, 40–50 years old, female

For some participants, this advocacy role became important, not only in seeking pain management, but to their own wellbeing and sense of self.

“You get fobbed off until you actually put your own foot down, become educated yourself. And what I'm saying here is, to cut a long story short, self-advocacy is the only thing that saved my sanity and got me on track.” P20, 50–60 years old, male.

## Discussion

Our findings suggest that for this sample of Australians with CNCP, the process of accessing health services for CNCP in Australia was complex and onerous, explaining, at least in part, why utilisation of evidence-based non-pharmacological treatments and multidisciplinary services may be often variable [23–25]. Participants faced barriers with almost every dimension of health care accessibility, from identification of health care needs and potentially helpful services, through to the ability to meaningfully engage with services and have needs met. In addition, we offer insight into how people may turn to self-advocacy in response to encountering barriers. While the rationale for this study was perceived low use of appropriate health care services by this population, participants reported high lifetime use of services, including 22 participants reporting pain specialist or pain service attendance. The experiences and range of barriers reported suggests that participants are accessing services, but encountering considerable obstacles to do so, highlighting the limitations of simply reporting health service utilisation as a measure of accessibility [34].

In a meta-ethnography, Toye et al. [37] described the experience of living with chronic musculoskeletal pain as an ongoing adversarial struggle against body and self, as well as the health system, and this struggle was illustrated in our study. Although previous studies have described skepticism about non-pharmacological treatments as a barrier to use [38–40], this was not evident in our study. Rather, despite repeated unsuccessful attempts to have needs met by the health system, most participants expressed willingness to continue exploring treatments and services that might offer relief. In this way, they demonstrated what Campbell and Guy [41] described as a ‘*tenacity rather than acquiescence’*, as well as the ambivalence identified by Toye et al. [37]: participants felt let down by the health system, yet compelled to continue engaging with said system.

Underpinning our findings is the continuing dominance of the traditional biomedical model of health and the over-medicalisation of pain. Although the biopsychosocial model was introduced in the 1970’s [6, 7], and is generally endorsed as the preferred model of pain management today [8, 9], clashes between the biomedical and biopsychosocial paradigms remain common in clinical training and practice [42, 43]. The biomedical model’s reductionist view of illness, and emphasis on diagnosis [44], can be observed in the experiences participants reported when initially seeking care. Like most Australians [45], participants approached GPs for non-emergent health problems. Our findings highlight the critical role primary care physicians can play in the immediate and ongoing management of CNCP, as well as how passive and active obstruction by doctors can hinder effective pain management. Despite increasing emphasis on patient participation and shared decision-making in care [46], participants who presented their own research to clinicians were often met with resistance, potentially due to their deviation from the traditional biomedical role of ‘*compliant patient*’, and their challenge to the role of the doctor as ‘*authoritative expert’* [44].

Some participants described engaging in behaviours which could be described as active self-management, which has been associated with reducing pain severity, pain catastrophising, and associated negative beliefs [47, 48]. However, participants described developing self-management strategies in response to the physical and emotional toll of attempting to access services, and often these strategies were accompanied by partial or complete withdrawal from the healthcare system. Self-management practices should be emphasised and taught to people with CNCP as part of routine pain management, rather than being left to develop in response to negative experiences, and these findings support calls for improved clinician training in contemporary pain management founded on the biopsychosocial health model [27].

External barriers to service use such as cost and geographical access are well described in the literature [39, 49–51], and our findings support assertions that current service provision models do not support best-practice pain management in Australia [27, 52]. Many participants were unaware of existing mechanisms to access subsidised care, despite long histories of health system engagement, echoing findings from a survey of 2233 Australians with CNCP in which one-quarter reported being unaware of these arrangements [53]. Participants in our study who used these arrangements agreed that subsidies were insufficient for CNCP, which often involves multiple chronic conditions requiring complex management, supporting calls for increased subsidy to be made available [52]. Waiting times for service access described here also support suggestions that demand for pain services significantly outweighs supply [2, 22, 27]. With evidence suggesting that significant deteriorations in health-related quality of life may occur in the initial six to twelve-months from referral to treatment access [54], there is a clear need to address systems-level factors impeding the accessibility of services for pain management, including increased investment into public pain services [27].

The variability reported in the management offered by different services of the same name highlights that the breadth and quality of treatments provided by a given service is not guaranteed, echoing European findings [55, 56]. In a review of Australian pain services, Hogg et al. [22] identified disparities in geographical distribution of public services offering pain management programs, suggesting that being in close proximity to a pain service does not guarantee proximity to multidisciplinary management. Services which appear similar on paper may offer different treatments in practice. For example, private pain clinics in Australia perform significantly more interventional procedures than public clinics, and group pain management programs offered across the country range from two to 120 h in duration [22]. Referral to services which are financially or geographically accessible, or simply more well-known to referring clinicians, may result in people receiving management which is not individualized or appropriate, leading to disengagement, inadequately treated pain, and disillusionment with services in the future. Similarly, patients who are referred to practitioners without training or experience in managing CNCP may receive treatments which are ineffective or harmful. These findings support the need for benchmarking and outcome reporting across pain services, and the development of primary care pathways so that people are referred to appropriate services most based on their needs.

### Strengths and limitations

Participants in this study accessed a range of treatments and services for CNCP and were well placed to provide insight into their experiences of accessing such services. In-depth interviewing allowing generation of rich data, and use of a framework conceptualizing access beyond service utilisation, allowed experiences of service accessibility to be fully explored [34]. Undertaking telephone interviews facilitated recruitment of participants from across Australia, including regional areas. Participants represented a spectrum of age ranges and lived with a range of conditions, resulting in findings which may be transferable to many people living with CNCP. Several strategies were employed to achieve a rigorous study and contribute to valid findings [57]. Piloting of the interview guide and the conduct and analysis of interviews by a researcher with training and skills in qualitative methods enhanced the credibility of findings. Use of a detailed study protocol developed by researchers with expertise in pain management, health services research, and qualitative research methodologies, and data management including maintenance of an audit trail enhanced the dependability of findings. Reflexivity was discussed at team meetings, with team members bringing differing perspectives to interpretation of the data. The interviewer’s memos and interview notes were triangulated with interview transcripts to increase the confirmability of findings.

This self-selected sample included participants recruited from consumer group websites, leading to a sample whose engagement with the health system may not be wholly representative of people with CNCP (and indeed, would likely have been greater). The participant group was predominately female, which may have implications for the transferability of findings to men. Participants described few experiences relating to the ‘Acceptability’ dimension of seeking care within cultural and societal contexts [34], potentially indicating a lack of sociocultural barriers to service access in Australia. However, we did not aim to specifically recruit sub-populations who might experience unique barriers and the opportunity for people from culturally and linguistically diverse backgrounds to participate was limited by undertaking interviews in English. Our ability to explore cultural factors associated with accessibility was therefore limited and findings may not be transferable to different cultural settings. Similarly, as codes were generated and mapped to Levesque’s framework a posteriori, participants were not asked specific questions about acceptability of seeking care. Regular team discussions were held throughout analysis to explore the differing perspectives and insights brought by team members, with the development of themes and concepts agreed upon by all team members; however, further insights may have been gained through independent double-coding, participant debriefing, or member checking of a selection of transcripts [57].

### Implications for future research and practice

Our findings demonstrate that improving health service accessibility for CNCP requires more than one solution. To bridge evidence-to-practice gaps and improve equitable access to evidence-based care, significant shifts are required in the way pain management is designed, funded, and delivered. There is a need to address deficiencies in practitioner education and to further support GPs, specialists, and allied health practitioners to deliver high-quality management based on contemporary understandings of pain and health. There is also a need to improve service provision, particularly in regional areas, and to expand current and establish new pain services, requiring significant support and investment from public and private service providers. Future areas of research should focus on sub-populations with unique needs, as well as practitioner perspectives of barriers to CNCP management provision.

While recruitment and interviews were conducted during the midst of the COVID-19 global pandemic, the study was conceived of prior to the pandemic and the focus was on understanding each participant’s overall treatment journey. As such, we did not seek to specifically understand the impact of the pandemic on access to health care services. Future research is required to understand how access to health services and ongoing CNCP management was affected by the considerable disruptions to health care systems and restriction of non-essential activities brought about by this global event.

## Conclusions

In this qualitative study of 26 Australians living with CNCP, barriers existed at almost every dimension of health care accessibility, from identification of health care needs, through to engagement with services and the ability to have needs addressed. Themes were underpinned by continuing overreliance on traditional biomedical models of health and systemic issues which make accessing health services for pain management difficult. With CNCP increasing in prevalence, and associated with enormous burden of disease, there is a clear need to address fundamental problems with health service accessibility for CNCP.

## Supplementary Information

**Additional file 1.** Semi-Structured Interview Guide.

**Additional file 2.** Participant Experiences Of Health Care Accessibility: Codes And Themes Mapped To Levesque’s Framework.

## Data Availability

The datasets generated and/or analysed during the current study are not publicly available due to privacy or ethical restrictions, but are available from the corresponding author on reasonable request.

## Abbreviations

CNCP: Chronic non-cancer pain
GP: General practitioner
