# Supplementary material for: “Frustrated with the whole system”: a qualitative framework analysis of the issues faced by people accessing health services for chronic pain
Source: BMC Health Serv Res. 2022 Dec 31;22:1603. doi: 10.1186/s12913-022-08946-8 (PMC9803895; doi:10.1186/s12913-022-08946-8)
Supplement: Supplementary file 1 — Additional file 1. Semi-Structured Interview Guide. [file 12913_2022_8946_MOESM1_ESM.docx]

**SUPPLEMENTARY INFORMATION FILE 1: SEMI-STRUCTURED INTERVIEW GUIDE**

Current pain condition/s

To begin with, I would like to learn a little about you and your pain condition.

1. Can you briefly tell me a little bit about your pain condition?

2. How do you currently manage your pain condition? *(Prompt: Do you currently/have you previously discussed your pain condition with a GP or other medical professional?)*

3. How does having chronic pain affect your day-to-day life?

Health service use

There are many different strategies for the management of chronic pain. Some of these include health services such as physiotherapy or psychological therapies.

5. Can you tell me about your experiences with other services such as physiotherapy for your pain?

6. There are also specialised pain management programs and pain clinics that can help people living with chronic pain. Have you ever accessed these types of services?

7. If yes to services: How did you learn about this service? How effective did you find these services? Have you experienced any barriers or trouble accessing these services? (If yes, did these barriers stop you from using these services? How did you overcome these barriers? How do you think your engagement with services would change if barriers such as cost were removed?)

9. If no to using services: Have these services ever been discussed or recommended by your GP or another health professional? What was the reason you did not access these services*?* (How do you think your engagement with services would change if those barriers were removed?)

11. If never heard of or considered: Would these services be of interest to you if your GP recommended or offered to provide a referral? Can you see any other barriers to using these services? (How do you think your engagement with services would change if barriers such were removed?)

12. Based on the discussion we’ve had today about services do you feel like your doctor provided enough information to you about the services available to you for pain? What other information would you like to receive or be given?

Experiences and recommendations

I’d like to ask some questions about your experiences receiving treatment for chronic pain in general:

13. How satisfied are you with how your pain is managed by your healthcare provider/s? Are your expectations for pain management being met?

14. Do you think that there have been any changes in how chronic pain is treated? (Can you describe this change you’ve experienced? Is this a good or bad change?)

15. Are there any experiences, either positive or negative, that you would like to share about accessing health services for chronic pain?

16. What recommendations would you make to your health care providers to best help you deal with your chronic pain?

17. Is there anything else you’d like to share today?
