# Supplementary material for: “Frustrated with the whole system”: a qualitative framework analysis of the issues faced by people accessing health services for chronic pain
Source: BMC Health Serv Res. 2022 Dec 31;22:1603. doi: 10.1186/s12913-022-08946-8 (PMC9803895; doi:10.1186/s12913-022-08946-8)
Supplement: Supplementary file 2 — Additional file 2. Participant Experiences Of Health Care Accessibility: Codes And Themes Mapped To Levesque’s Framework. [file 12913_2022_8946_MOESM2_ESM.docx]

**SUPPLEMENTARY FILE 2: PARTICIPANT EXPERIENCES OF HEALTH CARE ACCESSIBILITY: CODES AND THEMES MAPPED TO LEVESQUE’S FRAMEWORK**

| Codes generated | Initial themes identified | Themes mapped to Levesque’s dimensions | Final themes defined |
| --- | --- | --- | --- |
| - Seeking diagnosis - Seeking treatment - Seeking information - Seeking referrals | Seeking care | **Approachability**  **/**  **Ability to identify**  Both health care needs, and the existence and availability of services that may address these needs, can be identified | **GP as guide or gatekeeper**  People expect GPs to be the touchpoint between them and the system.  GPs play a vital role in linking people with services but can be passively and actively obstructive.  Having an unclear diagnosis exacerbates these issues. |
| - GP role - GP as leader of team - GP as barrier - GP knowledge | GP role |  |  |
| - Need for diagnosis - Unclear/no diagnosis - Misdiagnosis | Diagnosis |  |  |
| - Information about condition - Information about services - Sources of information - Own research | Information provision |  |  |
|  |  | **Acceptability**  **/**  **Ability to seek**  It is culturally and socially acceptable to seek and accept care from services |  |
| - Services available in the area - Access to transport - Travel required - Accessibility of physical space | Geographic access | **Availability and Accommodation**  **/**  **Ability to reach**  Services can be physically reached in a timely manner. | **Outside of my control: external barriers limiting access**  Geographical access is an issue even in urban areas. Many people don’t have access to transport.  Waiting times are considerable for public and private services.  Costs are prohibitive and not always direct. Subsidies are insufficient. |
| - Ability to get referral - Denied service - Waiting lists | Timely access |  |  |
| - Private health insurance - Out-of-pocket costs - Medicare subsidies - Compensation schemes - Unable to pay | Direct costs | **Affordability**  **/**  **Ability to pay**  Services are affordable, from both a financial and time point of view |  |
| - Time - Emotional cost, fatigue | Indirect costs |  |  |
| - Coordination of care - Provider information sharing - Individualised/personalised care - Flexibility of care | Coordinated and individualised care | **Appropriateness**  **/**  **Ability to engage**  The fit between services and need, including the quality of services and the ability of services to address health needs | **Services aren’t always good enough: there is wide variation in services and quality**  Services received are not always as described; people expect one thing and receive another.  Wide variation in offerings w/in ‘same’ category in what is offered, care quality, support given.  Providers without CNCP experience can be ineffective or harmful. |
| - Expectations of services - Expectations of outcomes - Past experiences - Perceptions of services | Treatment expectations and goals |  |  |
| - Provider training - Provider experience with CNCP | Provider knowledge |  |  |
| - Satisfaction with care - Persisting unmet needs | Satisfaction |  |  |
| - Emotional impact - Disengagement - Avoidance | Impact of access issues |  | **Leading my own pain management: the response to accessibility issues**  Inaccessibility and dissatisfaction lead to disengagement from services.  After time, people re-engage, armed with new research and assertiveness, taking charge of care. |
| - Previous learnings - Own routine - Doing nothing | Self-management |  |  |
| - Taking charge - Having to advocate - Educating providers | Self-advocacy |  |  |
